# Video-based multi-target multi-camera tracking for postoperative phase recognition

**DOI:** 10.1007/s11548-025-03344-x

**Published:** 2025-04-12

**Authors:** Franziska Jurosch, Janik Zeller, Lars Wagner, Ege Özsoy, Alissa Jell, Sven Kolb, Dirk Wilhelm

**Affiliations:** 1 Technical University of Munich, School of Medicine and Health, TUM University Hospital, Research Group MITI, Munich, Germany; 2 Technical University of Munich, School of Computation, Information and Technology, Chair of Computer Aided Medical Procedures, Munich, Germany; 3 Technical University of Munich, School of Medicine and Health, TUM University Hospital, Department of Surgery, Munich, Germany

**Keywords:** Patient tracking, Surgical workflow analysis, Surgical data science

## Abstract

**Purpose:**

Deep learning methods are commonly used to generate context understanding to support surgeons and medical professionals. By expanding the current focus beyond the operating room (OR) to postoperative workflows, new forms of assistance are possible. In this article, we propose a novel multi-target multi-camera tracking (MTMCT) architecture for postoperative phase recognition, location tracking, and automatic timestamp generation.

**Methods:**

Three RGB cameras were used to create a multi-camera data set containing 19 reenacted postoperative patient flows. Patients and beds were annotated and used to train the custom MTMCT architecture. It includes bed and patient tracking for each camera and a postoperative patient state module to provide the postoperative phase, current location of the patient, and automatically generated timestamps.

**Results:**

The architecture demonstrates robust performance for single- and multi-patient scenarios by embedding medical domain-specific knowledge. In multi-patient scenarios, the state machine representing the postoperative phases has a traversal accuracy of $84.9 \pm 6.0\%$, $91.4 \pm 1.5\%$ of timestamps are generated correctly, and the patient tracking IDF1 reaches $92.0 \pm 3.6\%$. Comparative experiments show the effectiveness of using AFLink for matching partial trajectories in postoperative settings.

**Conclusion:**

As our approach shows promising results, it lays the foundation for real-time surgeon support, enhancing clinical documentation and ultimately improving patient care.

## Introduction

Automatic documentation and optimization of clinical processes, as well as intelligent assistance due to context awareness, is very important in hospitals, especially considering the increasing staff shortage [1, 2]. For surgical treatments, deep learning-based networks have achieved promising results in accurately recognizing surgical phases [3], understanding whole scenes, and predicting clinical roles [4] to generate context understanding. Furthermore, the gained information can be used to document and optimize surgical processes.

Current research and technology only focus on supporting the actual surgical procedure, concluding once the surgery is finished [5]. However, the end of a surgery does not signify the end of the patient’s journey and hospital stay. Postoperative care and monitoring in recovery are equally and sometimes even more important for patient outcome [6], but remain underexplored with regard to phase recognition and creation of context awareness. The patient’s current location information can be used to automatically adjust device parameters, and automatic timestamp generation can reduce the documentation workload for clinical staff. Although sensor-based tracking solutions to locate medical equipment and people already exist, they are currently not widely used [7]. Camera-based approaches bring several other advantages, as the images can be used for multiple purposes to enhance overall patient care and support clinical staff. In an autonomous patient bed scenario, external cameras could, for instance, detect a patient’s protruding extremities, helping these beds to navigate without injuring patients. Furthermore, surgeons could check their patient’s postoperative state remotely and obtain a visual impression through the camera feed after leaving the operating room (OR). Finally, as initial research has already demonstrated, camera images could also be used to monitor patients’ vital parameters [8].

As a first step in this direction, we present a custom video-based multi-target multi-camera tracking (MTMCT) setup to detect postoperative phases, automatically generate timestamps of room changes, and provide the location of the patient during the postoperative process. This article focuses on the postoperative patient flow from the end of surgery to the recovery unit or intensive care unit (ICU) in particular.

## Related work

The aim of **video-based multi-object tracking (MOT)** is to accurately identify, locate, and track multiple objects or people in video sequences. This process is carried out without any prior knowledge of the object’s position or characteristics. [9, 10] The process of MOT typically involves four steps [9]: First, *object detection* is performed to identify the presence of objects or people within a video frame. Second, with *feature extraction or motion prediction*, additional information on detected objects or people is gathered. Third, a *similarity score* of the objects or people is calculated. Finally, the objects or people are *associated* with each other across the frames by assigning unique identifiers (IDs) to allow tracking over time. Established online MOT methods include DeepSORT [11] and ByteTrack [12], which use only present or past information to generate tracks [13]. ByteTrack shows competitive performance in tracking vehicles and pedestrians, is robust to occlusions, and allows videos to be processed at 30 frames per second (fps) [12].

In comparison to MOT, **multi-target multi-camera tracking (MTMCT)** uses multiple camera signals as input. This offers advantages, such as compensating for line-of-sight problems within one room or expanding the tracking radius of objects, but also increases the problem’s complexity, as data fusion and association between the different cameras become necessary. Furthermore, synchronizing the cameras and dealing with changes in camera viewpoints is essential [13]. Solving multi-view problems can be addressed using either a global solution or a two-step approach, where local tracks are generated for each camera by a MOT algorithm individually and merged into global tracks afterward [14]. Examples of online two-step algorithms are TRACTA [14] and DyGLIP [15]. However, as research on MTMCT mainly focuses on two domains (tracking vehicles in traffic and tracking pedestrians), and open-source MTMCT data sets focus on non-hospital environments [13], these solutions are not optimized for postoperative phase recognition.

In this paper, we therefore present a custom MTMCT architecture to recognize postoperative phases. Our solution is further capable of automatically generating timestamps and recording the patient’s path in the OR wing. Our contributions are: 1) We created the first data set containing postoperative processes. 2) We propose a novel MTMCT architecture for the postoperative workflow including medical domain knowledge. 3) We evaluate our approach through comprehensive experiments.

## Methods

### Task formulation

**Fig. 1:**
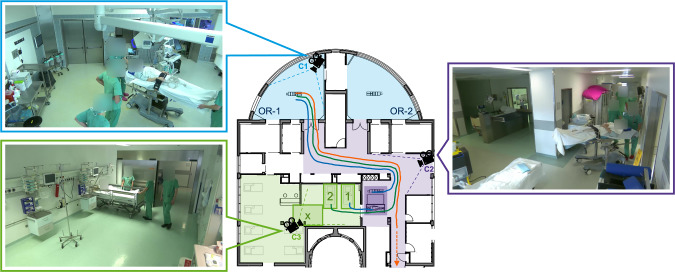
Overview of the clinical setup and camera positions during the recordings at TUM University Hospital. On the floor plan [16], the two operating rooms (OR-1 and OR-2) are colored blue, the recovery unit is green, and the hallway is purple. The final bed positions in the recovery unit are marked 1 and 2. For each scenario, an example trajectory is given. The trajectory from the OR via the transfer unit (dark purple) is dark blue (Scenario 1), direct transfer to the recovery unit is dark green (Scenario 2), and transfer to the ICU is orange (Scenario 3). Furthermore, camera positions, as well as their field of view, are shown on the floor plan. For each camera, an exemplary image is given

As this article focuses on the postoperative patient flow to the recovery unit or ICU, we consider three distinct scenarios for the postoperative patient flow in our clinical setup (see Fig. 1). Scenario 1 (S1) is the most commonly encountered situation. After surgery, the patient is moved to the transfer unit in the hallway while lying on the OR table. There, the patient is transferred into the designated patient bed and brought to the recovery unit. The transfer unit is used to reduce physical strain on clinical staff while moving the patient’s body. In Scenario 2 (S2), the patient is directly transferred to the patient bed in the OR and then moved to the recovery unit. Scenario 3 (S3) describes the case of an intensive care patient. The patient is transferred to an ICU bed in the OR and transported to the ICU for further care.

For the postoperative phase recognition, we define three main phases: *Transport*, *Patient transfer* and *Recovery*. While the *Transport* phase refers to the movement of the patient to another location, whether on the OR table or a care bed, the phase *Patient transfer* refers to the relocation of the patient from the OR table to a designated care bed. *Recovery* describes the patient’s location in the recovery unit under postprocedural observation. Depending on the scenario, the order of these phases may vary.

In this article, we aim to detect these three postoperative phases by multi-camera-based path tracking of patients, beds and OR tables. Furthermore, we want to derive time information of room changes to facilitate manual timestamp documentation. Lastly, we intend to obtain real-time location data to track the patient’s position during the whole process.

### Data set

To train a model for postoperative phase recognition, we created our own data set at TUM University Hospital. The data set consists of 19 postoperative patient flows (8$\times$S1, 6$\times$S2, 5$\times$S3), reenacted by physicians and medical volunteers. Roles and people were swapped during the video recordings, and their appearances varied. Empty beds and other medical equipment were moved along plausible trajectories to enhance object diversity. To simulate waiting times during patient transport, we included crossing trajectories of these objects and the transported patient. Finally, people were shown walking by, entering other rooms, and talking to incorporate realistic scenes into the data set. The scenarios were filmed using three RGB cameras (2$\times$Panasonic AW-UE100 and 1$\times$Panasonic AW-UE80) at a resolution of 1080p with 50 fps. The camera positions and angles are marked in Fig. 1. The camera in the OR (C1) views the surgical field and exit of the OR, while the hallway camera (C2) views the hallway to the ORs as well as the transfer unit. The camera in the recovery unit (C3) provides a good view of the entrance as well as bed positions 1 and 2. Bed position *x* represents a transfer to any other bed position in the recovery unit but was not considered in these 19 cases. The video recordings of all cameras were annotated at 25 fps with bounding boxes for the object categories: OR table, care bed, and patient. The category care bed includes both ICU and patient beds. In total, the data set contains more than 150,000 frames.

### Network architecture

**Fig. 2:**
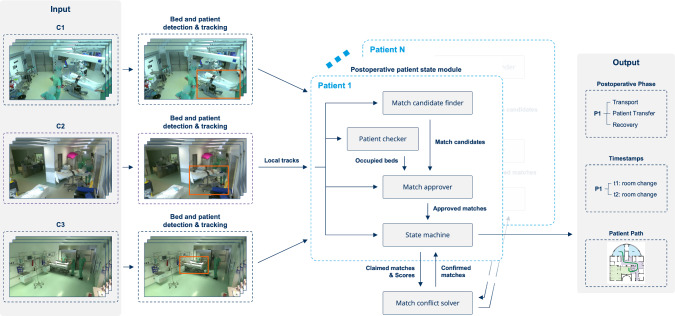
Overview of our proposed MTMCT architecture for postoperative phase recognition. Detection and tracking of OR tables, care beds, and patients are conducted camera-wise. The local tracks are processed into global tracks within the postoperative patient state module, which outputs the postoperative phase, timestamps, and tracking path for each patient

Our proposed network architecture (Fig. 2) incorporates the multi-object tracker ByteTrack [12], utilizing the YOLOv8 [17] object detection model as the foundation for detecting and tracking OR tables, care beds, and patients. This is followed by several postoperative patient state modules, each containing a patient checker, match candidate finder, match approver, and a state machine (SM) that returns the postoperative phase and associated timestamps. The global match conflict solver resolves conflicts between individual postoperative patient state modules.

#### Multi-object tracker and data set extension

To generate local tracks of OR tables, care beds, and patients for each camera, we use YOLOv8 for object detection and ByteTrack for the tracking task. To prevent overfitting due to oversaturation of training data with similar frames, we reduce the frame rate in sections of the videos where objects remain in static positions. The resulting tracks in the 2D image space are then projected to 3D positions in the operating wing as shown in Fig. 1 using the intrinsic and the extrinsic camera matrix in the world coordinate space.

As the recorded data set only includes single-patient scenarios, we expanded the data set with synthetically generated tracks of patients leaving OR-1 and OR-2 to simulate multi-patient scenarios based on these projected 3D trajectories. The synthetic tracks include trajectories with patients moving to various recovery positions or the ICU. We incorporated realistic variations in speed, random accelerations, and Gaussian noise to replicate real-world conditions. Additionally, missing detections and changing track IDs were introduced to simulate challenges such as occlusions and tracking errors. In total, 252 synthetic patient flows were generated.

#### Postoperative patient state module

As common multi-camera trackers are primarily used for pedestrians and vehicles in traffic, we cannot reuse their domain-specific solutions for matching local paths in our scenarios. Therefore, we exploit the domain-specific knowledge available in our medical environment to develop a customized tracking solution in postoperative settings. This involves utilizing patient-specific data and characteristics within our proposed postoperative patient state module. The components of the module are briefly presented below.

*Patient checker* Since patients are usually covered up and wear standardized clothing, including a hairnet, consistent tracking is challenging. Usually, only parts of their face, body outline, or arms are visible, and they are often partially occluded by medical staff or equipment. However, since patients remain on an OR table or in their care bed throughout their transport in the operating wing, we focus on tracking these instead. To ensure that a bed is occupied, we check if the patient’s bounding box overlaps with the bed’s bounding box in each frame. The thresholds for the intersection of two bounding boxes divided by the patients’ bounding box area for care beds $IoU_\text {pat,cb}$ as well as for OR tables $IoU_\text {pat,ort}$ are hyperparameters. Beds are considered empty until a patient is detected at least once.

*Match candidate finder* This component identifies pairs of partial tracks, called match candidates, that could belong to the same bed. Pairs must consist of the same bed type to become a match candidate. Tracks from cameras covering unconnected rooms are excluded. A spatial distance limit $d_\text {max}$ is imposed for tracks at the same time, with an allowance for temporal gaps based on a maximum patient speed of $v_\text {max}$. Once a match candidate pair is created, the system continues updating trajectories with subsequent detections to refine the match while minimizing unnecessary computations.

*Match approver* The match approver combines the information of the patient checker and match candidate finder to validate and approve potential matches, ensuring that only tracks consistent with the detected bed movements and occupations are accepted. It waits until the new track in a candidate pair has at least $N_\text {min}$ data points, filtering out short tracks and ensuring sufficient information for accurate similarity evaluation. Spatially and temporally non-overlapping tracks are approved by AFLink [18] if the similarity score exceeds the similarity threshold $\tau _\text {sim}$. AFLink performs data association without relying on appearance information, using only trajectory data in the 3D image space, offering a faster alternative to traditional MOT methods that combine Kalman filters with complex feature extractors for appearance matching.

**Fig. 3:**
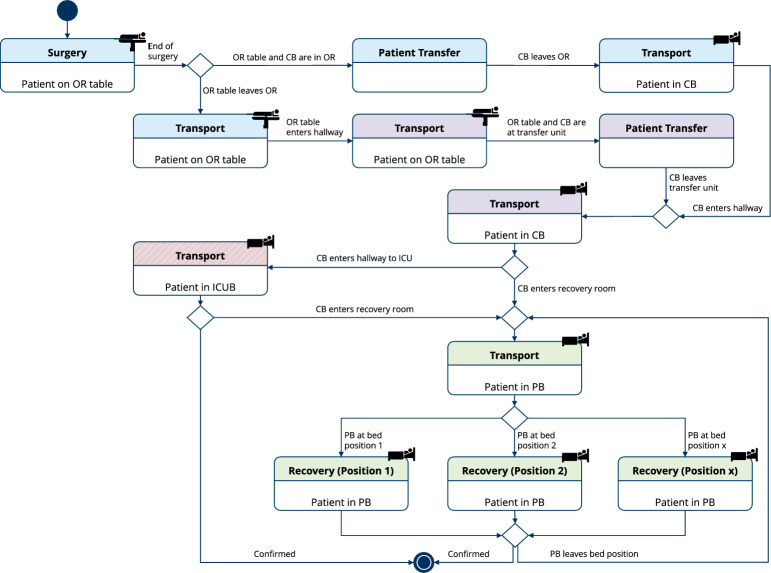
State machine of the postoperative phases. The states *Transport*, *Patient transfer*, and *Recovery* are color-coded for the OR (blue), hallway (purple), recovery unit (green), and ICU (red). The distinction whether the patient is in a patient bed (PB) or an ICU bed (ICUB) is made by detecting the pathway of the care bed (CB)

*State machine* The match approver feeds its approved matches to the state machine (SM), which is shown in Fig. 3. The states represent the phases of the postoperative process, and transitions are allowed based on new detections that belong to the current track. Beside the patient path, timestamps are created based on the moment the patient’s trajectory fulfills the condition of the corresponding state transition by entering a new area.

*Match conflict solver* The match conflict solver ensures that the same partial track is not assigned to multiple patients by evaluating claimed matches from each patient’s SM. If there is no overlap, all claims are confirmed, and SMs are updated to prevent further conflicts. When multiple patients claim the same track, the solver resets the affected SMs and resolves the conflict by considering their states, favoring patients further along in their state transitions or those not in a final state. If this does not resolve the issue, similarity scores based on AFLink are used, with the highest score determining the match. In rare cases of identical scores, the conflict is resolved by chance.

### Experimental setup

#### Model training

The YOLOv8 network was purely trained on real-world data, while synthetic data served to train the trajectory similarity network AFLink and parameter tuning of the patient state module. To facilitate 4-fold cross-validation for the object detector and tracker, the 19 real scenarios were divided into folds in the following way: Fold1 (2$\times$S1, 2$\times$S2, 1$\times$S3), Fold2 (2$\times$S1, 1$\times$S2, 1$\times$S3), Fold3 (2$\times$S1, 1$\times$S2, 1$\times$S3), and Fold4 (2$\times$S1, 1$\times$S2, 2$\times$S3). It was ensured that the patients included in each validation fold were not present in the training folds. The YOLOv8 network was trained for each camera for 120 epochs with a batch size of 16 on an NVIDIA RTX A4500 using PyTorch version 1.8. The hyperparameters for the training were based on the YOLOv8n default model with an input image width of 640p. The learning rate followed a linear schedule with a starting learning rate of 0.01 and a final learning rate change factor of 0.01. The optimizer was set to auto with a momentum of 0.937 and a weight decay of 0.0005. The IoU threshold for non-maximum suppression was 0.7.

To train AFLink on the synthetic data set, we used both clean and noisy 3D trajectories. Complete trajectories were split into partial trajectory pairs at points where tracking gaps occurred. Furthermore, trajectories were randomly divided at arbitrary positions, resulting in varying segment lengths. These partial trajectory pairs served as ground truth samples for conducting the initial AFLink training. The data were divided into equal sets of 63 distinct scenarios for a 4-fold cross-validation.

#### Evaluation metrics

To comprehensively measure the results, we report common object detection metrics such as mean average precision (mAP) as well as common tracking metrics such as precision, recall, IDF1, the multiple object tracking accuracy (MOTA), and the higher-order tracking accuracy (HOTA). To evaluate the performance regarding the phase recognition task, we provide the SM traversal accuracy, which represents the percentage of scenarios in which the SM is fully traversed correctly, meaning all phases of the postoperative process are accurately recognized for a patient. The timestamp accuracy measures the percentage of generated timestamps that are considered correct with a tolerance of 1 s. In addition, we provide the patient IDF1, measuring the patient tracking performance, whereby only patient object tracks are considered and bed tracks are ignored. We perform ablation tests with AFLink in the match conflict solver and match approver to assess its influence on the accuracy of trajectory matching and the effectiveness of conflict resolution.

**Table 1:** Evaluation metrics of the multi-object tracking for different cameras and object categories

		YOLOv8	ByteTrack				
	Object	mAP 50-95	Precision	Recall	IDF1	MOTA	HOTA
C1	OR table	$90.5 \pm 1.7$	$96.2 \pm 1.3$	$100 \pm 0.0$	$98.0 \pm 0.7$	$96.0 \pm 1.4$	$98.0 \pm 0.7$
	Care bed	$91.2 \pm 3.8$	$92.4 \pm 3.8$	$99.8 \pm 0.2$	$93.6 \pm 6.6$	$91.4 \pm 4.7$	$93.4 \pm 5.9$
	Patient	$70.0 \pm 4.5$	$94.0 \pm 2.1$	$88.0 \pm 3.7$	$65.6 \pm 10.5$	$82.0 \pm 2.4$	$68.9 \pm 7.6$
C2	OR table	$82.4 \pm 5.3$	$83.9 \pm 3.8$	$98.3 \pm 2.2$	$88.1 \pm 5.2$	$79.2 \pm 2.0$	$86.5 \pm 5.6$
	Care bed	$86.3 \pm 8.4$	$87.4 \pm 1.9$	$99.9 \pm 0.2$	$93.0 \pm 1.2$	$85.2 \pm 2.6$	$93.0 \pm 1.1$
	Patient	$47.1 \pm 4.9$	$57.5 \pm 6.9$	$63.1 \pm 17.6$	$30.6 \pm 7.8$	$14.9 \pm 17.6$	$35.0 \pm 8.5$
C3	Care bed	$94.3 \pm 3.3$	$94.2 \pm 6.2$	$99.6 \pm 0.9$	$95.5 \pm 6.3$	$93.1 \pm 8.3$	$95.7 \pm 5.5$
	Patient	$51.0 \pm 5.7$	$74.7 \pm 14.4$	$65.4 \pm 15.8$	$40.5 \pm 5.7$	$36.7 \pm 12.2$	$40.8 \pm 4.3$

The average metrics (%) with their respective standard deviations (±) are provided for each object category, based on 4-fold cross-validation in object detection and tracking. For ByteTrack, the score threshold for the first association is set to 0.5, the overall score threshold to 0.1, the buffer size to 30, and the match threshold to 0.8

## Results

### Multi-object tracking

In Table 1, we show detection and tracking results for the three cameras and three different object categories: OR table, care bed, and patient. Due to the small number of real-world scenarios, we applied a 4-fold cross-validation. In general, YOLOv8 shows good mAP, with the detection of patients being the most challenging, especially in the hallway and the recovery unit. ByteTrack generally works well for the object tracking task, especially for the OR table and care bed. Tracking the patient in the hallway (C2) and the recovery unit (C3), on the other hand, achieved the worst scores in comparison with the other object categories. The higher recall relative to precision for OR tables and care beds suggests that the models are sensitive.

### Postoperative phase recognition

Table 2 shows the results for the postoperative phase recognition task on the expanded data set with synthetically generated scenarios and the impact of utilizing AFLink in both the match conflict solver and match approver. We also performed a 4-fold cross-validation and provided the average metrics along with their respective standard deviation. The best results were obtained for $IoU_\text {pat,cb} = 0.99$, $IoU_\text {pat,ort}=0.8$, $d_\text {max}=1m$, $v_\text {max}=0.65\frac{m}{s}$, $N_\text {min}=20$, and $\tau _\text {sim}=0.05$.

In the single-patient scenarios, all phases are recognized correctly, and $97.1 \pm 2.3\%$ of the timestamps are accurate over all variations. An IDF1 score of $99.6 \pm 0.6\%$ is achieved, with a decline of $0.3\%$ when including AFLink in the match approver.

For multi-patient scenarios, using AFLink for conflict solving improves the results for all metrics. Furthermore, threshold matches via AFLink contribute to an additional improvement. Our complete method detects all phases correctly in $84.9 \pm 5.9\%$ of postoperative processes. The generated timestamps achieve an accuracy of $91.4 \pm 1.5\%$ with a transitional delay tolerance of 1 s. The patient tracking performance demonstrates robust results, with an IDF1 score of $92.0 \pm 3.6\%$, indicating reliable identification and tracking consistency.

**Table 2:** Evaluation metrics of our proposed architecture on postoperative phase recognition for single- and multi-patient scenarios

AFLink in match	AFLink in match	SM traversal	Timestamp	Patient
conflict solver	approver	accuracy	accuracy	IDF1
Single-patient scenarios				
✗	✗	$100 \pm 0$	$97.1 \pm 2.3$	$99.6 \pm 0.6$
✓	✗	$100 \pm 0$	$97.1 \pm 2.3$	$99.6 \pm 0.6$
✓	✓	$100 \pm 0$	$97.1 \pm 2.3$	$99.3 \pm 0.6$
Multi-patient scenarios				
✗	✗	$74.0 \pm 3.8$	$87.6 \pm 2.1$	$87.0 \pm 2.1$
✓	✗	$83.4 \pm 4.1$	$90.4 \pm 2.1$	$89.5 \pm 2.8$
✓	✓	$84.9 \pm 5.9$	$91.4 \pm 1.5$	$92.0 \pm 3.6$

Average metrics (%) with their respective standard deviations (±) are reported for a 4-fold cross-validation

## Discussion

Our proposed custom MTMCT architecture accomplishes a first step in the direction of camera-based postoperative workflow detection. It further allows automatic timestamp generation and location estimation, which can be used to automatically document the postoperative process or as context input for other assistive functions. The general object detection of YOLOv8 is good, although the detection of patients is challenging. The many occlusions of the patient due to medical personnel, the patient bed, the blanket, and the camera positioning explain this weak detection performance, especially in the hallway and recovery unit. Even the use of ByteTrack, which is known for its robustness against occlusions, did not completely solve the problem and the challenges in accurately tracking patients remain evident. In the future, optimizing camera positions and using multiple cameras per room are important factors to consider in order to minimize occlusions and therefore improve the tracking results.

Despite the partially low tracking performance on patients, the proposed architecture of the postoperative patient state module shows remarkable results for postoperative phase recognition by including medical domain knowledge. Ablation tests demonstrate the positive impact of using AFLink in the match approver and match conflict solver for multi-patient scenarios. Transition timestamps and location information could be provided with high accuracy.

However, some challenges must be addressed before our method can be used in daily clinical practice. Since the data set only includes 19 patient flows, it should be extended to improve robustness for other bed and patient appearances. Here, more cameras with overlapping camera views could be beneficial and multi-patient scenarios should be recorded. In addition, other object detection algorithms or segmentation methods could be tested to increase the reliability of patient tracking. To improve the match performance of partial tracks, additional similarity metrics should be considered. Moreover, the transferability and generalizability to other clinical setups should be investigated.

A camera-based approach for patient tracking and postoperative phase recognition has ethical implications. Of course, medical professionals and patients must be made aware of the use of cameras. Our approach aims to directly process live image streams and only store the outputs, such as timestamps, phases, and localization, making storage of video data unnecessary. By analyzing the video data on-site at the hospital and protecting it to the same extent as other sensitive patient data, unauthorized access can be prevented, which is essential for the clinical integration of such a system.

## Conclusion

In this article, we present a custom MTMCT architecture for postoperative phase recognition, automatic timestamp generation, and location estimation. For each camera, a MOT algorithm was trained on 19 reenacted postoperative patient flows containing more than 150,000 frames. We achieved good tracking results for the object categories OR table and care bed, and moderate tracking results for patients. Nevertheless, the overall architecture achieved remarkable results for postoperative phase recognition ($84.9 \pm 5.9\%$), timestamp generation ($91.4 \pm 1.5\%$), and patient localization ($92.0 \pm 3.6\%$) in multi-patient scenarios. Consequently, it provides a basis for real-time clinician support, improving clinical documentation and ultimately enhancing patient care. Beyond a continued optimization of this approach in the future, successful concepts from intraoperative cases should be extended to postoperative processes and entirely new approaches should be developed to fully exploit the benefits of camera-based solutions in postoperative settings.

## Data Availability

The data sets generated and analyzed during the current study are not publicly available due to restrictions related to privacy concerns for the research participants but are available from the corresponding author on reasonable request.

## Code Availability

The underlying code for this study is available at https://github.com/ResearchgroupMITI/PPT.
